# Comparison between Mechanical Properties and Structures of a Rolled and a 3D-Printed Stainless Steel

**DOI:** 10.3390/ma12233867

**Published:** 2019-11-23

**Authors:** Stefano Natali, Andrea Brotzu, Daniela Pilone

**Affiliations:** Dipartimento ICMA, Sapienza Università di Roma, Via Eudossiana 18, 00184 Roma, Italy; stefano.natali@uniroma1.it (S.N.); andrea.brotzu@uniroma1.it (A.B.)

**Keywords:** additive manufacturing, mechanical properties, selective laser melting

## Abstract

In this work selective laser melting was successfully utilized to produce 316 stainless steel bulk specimens. Although this technology provides many advantages compared to conventional shaping processes, little residual porosity may be a problem for some applications where high strength is required. The objective of this work was to determine, through data analysis, a mechanical and metallographic comparison between thin sheets made by using different manufacturing technologies: Cold rolling and additive manufacturing. This comparison was useful to understand whether it could be more advantageous to use the prototyping for new mechanical components. The results show that the additive manufactured steel, due to its microstructure, is characterized by a higher yield strength and by a lower elongation and ultimate tensile strength.

## 1. Introduction

Additive manufacturing is an advanced technology that allows to produce components having complex shapes. The material is added layer upon layer by using 3D design data. Metal additive manufacturing has attracted considerable interest in the industry to produce parts characterized by intricated structures. Unlike conventional casting and thermo-mechanical treatment, additive manufacturing allows to produce near-net shape components and then the alloy microstructure obtained with the production process usually remain unchanged [1,2]. It must also be stressed that material areas in different directions are subjected to different thermal cycles, resulting in anisotropic mechanical properties [3,4]. To date the relationship between the process parameters and mechanical properties of the produced components is not well known. Additive manufacturing is a flexible technique that can produce components having any geometry by using digital design data.

Stainless steels are one of the most widely investigated materials for selective laser melting. In fact, this process allows to produce both dense and porous stainless-steel components used in the aerospace and automotive industry [5].

By analyzing the data available in the literature, ferrous alloy components are usually produced by using Laser Powder-Bed (LPB) (also known as selective laser melting), Laser Powder-Fed (LPF) and Binder Jetting (BJ). By analyzing data relative to materials produced by means of LPF it seems that the ultimate tensile strength and yield strength of LPF components are mostly greater than the wrought components. This could be due to the higher cooling rate and grain refinement characterizing the additive manufacturing process. Experimental results also highlighted a lower elongation due probably to porosities and inclusions present in the material due to the selection of wrong parameters. LPF processed steels show lower elongation-to-failure values; this is probably due to porosity caused by the selected operative parameters.

Many papers available in the literature highlight the relationship between process parameters used in selective laser melting and mechanical properties of the alloy [6,7,8]. It has been found that microstructure and performance can be optimized by adjusting laser scanning paths and that a solid solution treatment determines the disappearance of the dendritic structure. A tailored laser scanning path and post-heat treatment are essential to produce a sound material having a uniform microstructure that has good mechanical behavior. Very recent papers investigated the relationship between the microstructure and mechanical properties of 316 stainless steels fabricated via additive manufacturing. It has been found that by using particular experimental conditions it is possible to obtain specimens characterized by high yield strength and ductility. The high yield strength has been attributed to both a high dislocation density and a special cellular and columnar substructure whose grain boundaries hinder dislocation movement [9,10,11]. On the other hand, twin generation seems to increase elongation [11].

The objective of this work is to determine, through data analysis, a mechanical and metallographic comparison between thin sheets made by using different manufacturing technologies: Cold rolling and additive manufacturing. This comparison will be useful to understand whether it could be more advantageous for the prototyping of new mechanical components. In fact, although the sheet-metal forming process is known to have excellent results in terms of surface finish and mechanical characteristics, it requires a long time for the realization of the die. Additive manufacturing technology could be an interesting alternative to sheet-metal forming because it would allow the realization of the same components in a shorter period of time.

## 2. Experimental

The 316L specimens were produced by using the patented LaserCUSING^®^ process. In this process, fine metal powders are melted locally by a high-energy fiber laser. The specimens were built up layer by layer (with a layer thickness of 20–80 μm) by lowering the bottom of the build chamber, applying more powder and then melting again.

The process parameters used to produce the specimens have been optimized to minimize material porosity. Process parameter values are confidential and cannot be disclosed. From the as-deposited specimens, metallographic samples were taken. Metallographic specimens were polished and etched. The microstructure of the alloys taken in different directions were observed by using both optical microscope and scanning electron microscope equipped with energy dispersion spectroscopy.

Tensile test specimens were cut from both rolled and printed 316 stainless steel in order to compare their mechanical properties. Tests have been carried out on both rolled and printed specimens having a thickness of 0.3, 0.5 and 0.8 mm.

**Figure 1:**
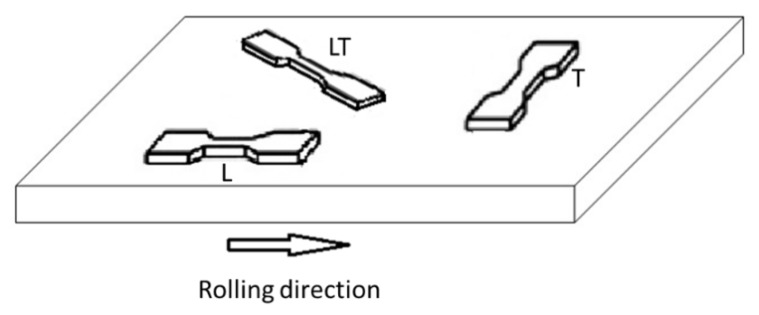
Sketch showing how tensile specimens were taken from the rolled plate.

In order to study the effect of the rolling direction on the material mechanical properties, tensile specimens were taken as shown in Figure 1. In fact, the rolling process produce the grains aligned in the rolling direction. This involves different mechanical behavior in different directions. For this reason, specimens were taken in different directions: 0°, 45° and 90° with respect to the rolling direction. For each selected direction and for each thickness we tested 3 specimens. As far as specimens prepared by means of additive manufacturing are concerned, 3 specimens for each considered thickness were tested.

Tensile tests were carried out for determining tensile strength, yield stress and elongation.

Microstructural analyses were carried out by means of SEM and optical microscopy on specimens after grinding and polishing and after electrochemical etching in a 10% aqueous oxalic acid bath. Microanalyses were carried out by means of energy dispersion spectroscopy.

## 3. Results and Discussion

**Figure 2:**
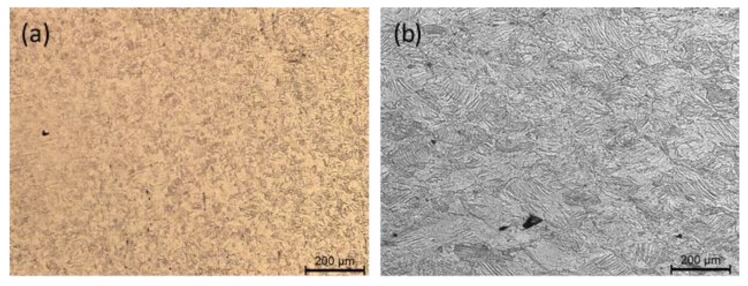
Optical micrographs showing the microstructure (planar view) of the rolled specimen (**a**) and of the 3D-printed specimen (**b**).

**Figure 3:**
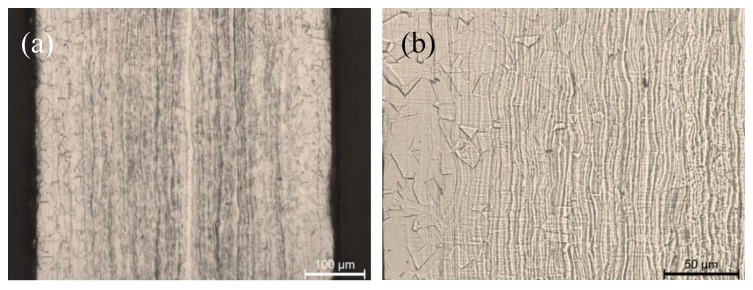
Optical micrographs showing the transversal section of the rolled specimen at lower (**a**) and higher (**b**) magnification.

Optical micrographs reported in Figure 2 show that both materials after etching show a very fine microstructure and that the one produced by means of additive manufacturing shows few cavities. By looking at the transversal section of steel sheets produced by cold rolling it is possible to observe the presence of shear bands due to plastic deformation located mainly at the sheet central part (Figure 3).

**Figure 4:**
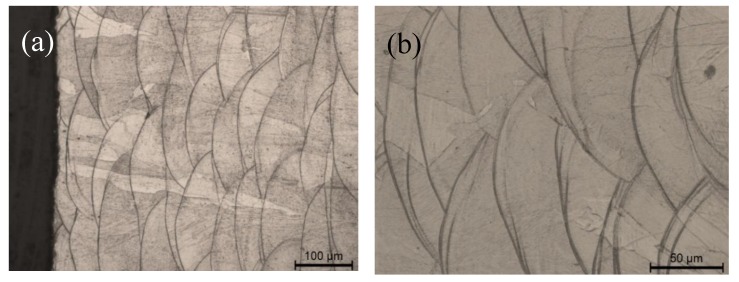
Optical micrograph showing the microstructure (transversal section) of the 3D-printed specimen at lower (**a**) and higher (**b**) magnification.

Figure 4 shows the transversal section of the specimen produced by means of additive manufacturing. The micrographs show the melting pools where the pool boundaries form circular arcs due to the energy distribution during laser melting. It can be observed that the pool boundaries are interlaced with each other and that there are no evident cavities. By looking at the microstructure, it can be observed that due to the thermal cycle the dendritic microstructure disappears and grains grow by crossing pool boundaries. The cross-sections of the samples show that the stainless-steel powder particles are completely fused together. Moreover, the laser tracks overlap so that each melting pool is bonded on the other pools surrounding it. Although the material produced by means of additive manufacturing is quite compact it is important to compare its properties with those of the rolled specimen.

**Figure 5:**
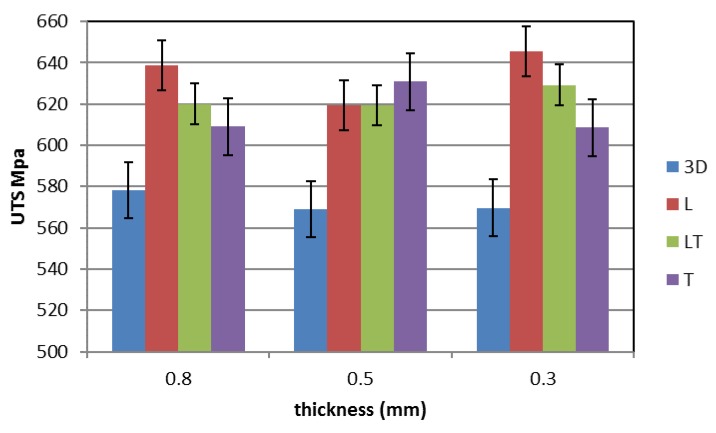
Ultimate tensile strength (UTS) of additive manufactured and cold-rolled 316 stainless steel for different specimen thicknesses.

**Figure 6:**
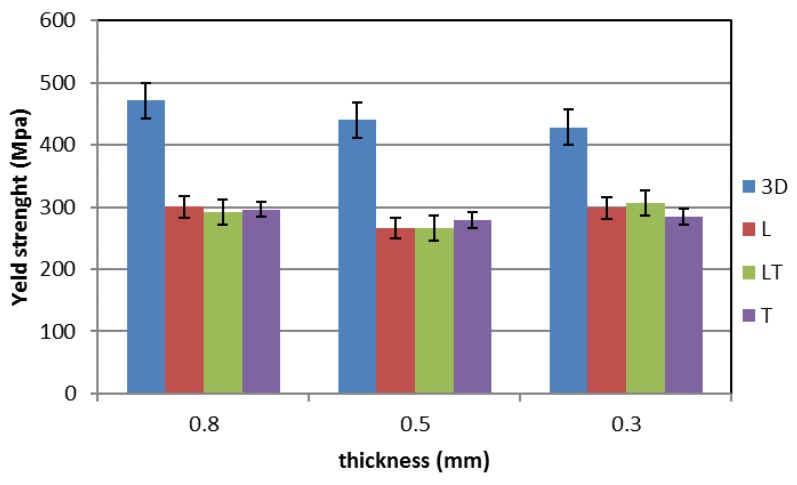
Yield strength of additive manufactured and cold-rolled 316 stainless steel for different specimen thicknesses.

**Figure 7:**
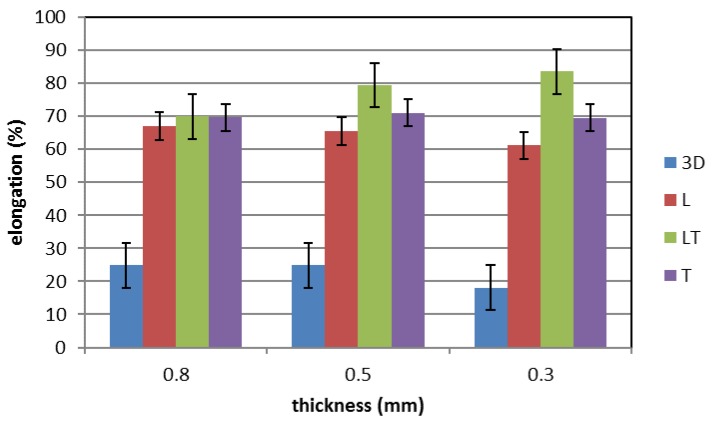
Elongation of additive manufactured and cold-rolled 316 stainless steel for different specimen thicknesses.

Figure 5, Figure 6 and Figure 7 show the mechanical properties of the additive manufactured material (blue) compared with those of the cold-rolled specimens taken in different directions (red—L longitudinal, green—45° LT and purple—T transversal) for the three different thicknesses tested. The mechanical property values are listed in Table 1. It can be observed that for the cold-rolled material the measured mechanical properties are independent from the considered direction. The ultimate tensile strength (UTS) is about 625 MPa (593–649 MPa, standard deviation 14.1), the Yield Strength is about 290 MPa (263–307 MPa, standard deviation 16.4) and the elongation is about 70% (58%–84%, standard deviation 6.7).

The mechanical properties of the steel produced by means of additive manufacturing are quite different from those of the cold-rolled steel. In fact, the UTS and the elongation are lower, while the yield stress is higher. In particular, a small reduction of the UTS (about 9%), a large reduction of the elongation (more than 60%) and a large increase in the yield stress (more than 45%) can be observed. Moreover, the influence of the thickness on the mechanical properties seems to be negligible. For the additive manufactured steel, the UTS is about 570 MPa (550–588 MPa, standard deviation 13.6), the yield strength is about 445 MPa (405–478 MPa, standard deviation 28.5) and the elongation is about 23% (11.6%–29.4%, standard deviation 6.8).

By analyzing these data it can be highlighted that for the additive manufactured steel, the microstructure, characterized by several as-cast melting pools and by a complex sub grain structure (Figure 4), produce a higher yield strength in comparison with the cold-rolled steel, as highlighted by several authors [10,11]. On the other hand, both UTS and elongation of the additive manufactured steel are lower than that of cold-rolled steel. In order to understand the reason for this peculiar behavior it must be highlighted that although most of the studies states benefits of additive manufacturing technology, there are still limitations concerning heterogeneity in the microstructure and mechanical properties [12,13,14]. Many authors discussed the effect of complex thermal cycles on the alloy microstructure. In addition, common defects such as pores, microshrinkage cavities and lack of fusion can change the mechanical behavior of the material. In this work the presence of few micro-discontinuities (Figure 2b) has become a source of stress concentration in the part and led to crack propagation and failure. This can justify why UTS and elongation of the additive manufactured steel are lower than the ones of the rolled steel. Obviously, those mechanical properties could be improved by checking the powder’s quality and process parameters. As already stressed in the literature [13] the discrepancy among data coming from different studies may be due to the complex microstructure and defects characterizing additive manufactured parts.

**Figure 8:**
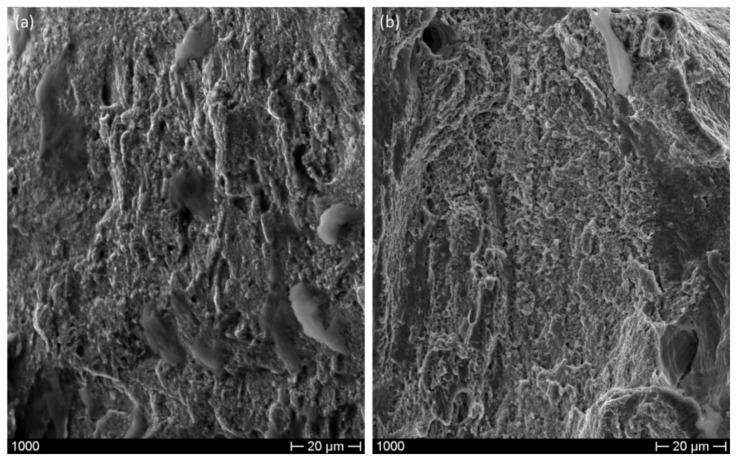
SEM micrograph showing the fracture surface of 3D-printed specimens having a thickness of 0.3 mm (**a**) and 0.8 mm (**b**).

**Figure 9:**
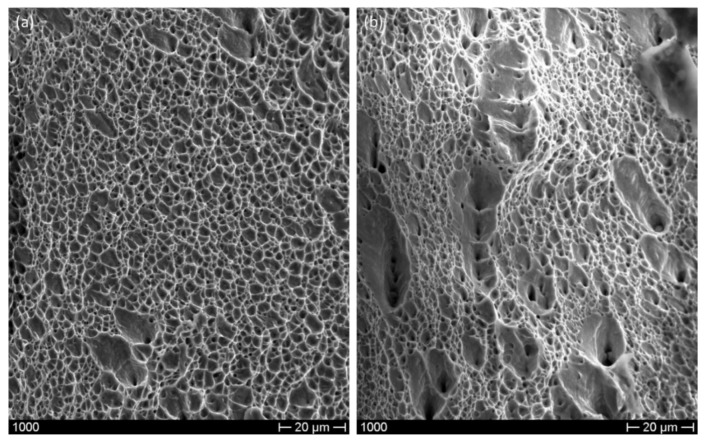
SEM micrograph showing the fracture surface of L rolled specimen (**a**) and T rolled specimen (**b**).

In order to understand the mechanical behavior of the tested materials it is important to analyze the fracture surfaces. Figure 8 shows that the additive manufactured material has a ductile behavior. In fact, the steel plastic deformation is apparent, and in some areas dimples can be observed. The rolled steel (Figure 9), characterized by a very high elongation, has fracture surfaces characterized by dimples. Figure 9b shows that the T tensile specimen shows bigger cavities due to the decohesion of shear bands.

## 4. Conclusions

The objective of this work was to perform a comparison between the mechanical behavior of thin sheets made by using cold rolling and additive manufacturing. Although the sheet-metal forming process is known to have excellent results in terms of surface finish and mechanical properties, it requires a long time for the realization of the die. The results highlighted that the additive manufactured steel is characterized by a higher yield strength and lower elongation although its behavior is still ductile. Moreover, its mechanical properties do not depend on the steel sheet thickness. Further studies on possible heat treatment could allow to obtain tailored properties.

## Figures and Tables

**Table 1:** Mechanical properties of 316 stainless steel produced by means of additive manufacturing (3D) and cold rolling. Three specimen thicknesses have been considered. Standard deviations are reported in parentheses.

Thickness (mm)	Ultimate Tensile Strength (MPa)				Yield Strength (MPa)				Elongation (%)			
	3D	L	L-T	T	3D	L	L-T	T	3D	L	L-T	T
0.8	578.1	638.5	620.0	609.2	471.7	300.1	291.8	296.2	24.9	67.0	70.0	69.7
	(10.9)	(1.7)	(2.0)	(2.6)	(7.8)	(3.4)	(10.8)	(11.0)	(7.3)	(1.8)	(3.6)	(4.9)
0.5	569.2	619.3	619.4	630.9	440.1	266.4	266.5	279.5	24.9	65.5	79.4	71.1
	(19.0)	(4.1)	(8.2)	(10.1)	(7.0)	(2.7)	(5.2)	(5.2)	(4.9)	(5.2)	(3.5)	(2.4)
0.3	569.7	645.5	629.2	608.5	428.3	298.7	306.3	284.0	18.1	61.2	83.6	69.5
	(12.6)	(3.0)	(11.8)	(12.9)	(8.9)	(1.7)	(0.4)	(2.9)	(9.1)	(2.3)	(0.1)	(5.5)

## References

[1] Fayazfar H., Salarian M., Rogalsky A., Sarker D., Russo P., Paserin V., Toyserkani E. (2018). A critical review of powder-based additive manufacturing of ferrous alloys: Process parameters, microstructure and mechanical properties. Mater. Des..

[2] Shamsaei N., Yadollahi A., Bian L., Thompson S.M. (2015). An overview of Direct Laser Deposition for additive manufacturing; Part II: Mechanical behavior, process parameter optimization and control. Addit. Manuf..

[3] Kobryn P.A., Semiatin S.L. Mechanical properties of laser-deposited Ti-6Al-4V. Proceedings of the Solid Freeform Fabrication Symposium.

[4] Yadollahi A., Shamsaei N., Thompson S.M., Elwany A., Bian L. Mechanical and Microstructural Properties of Selective Laser Melted 17-4 PH Stainless Steel. Proceedings of the ASME 2015 International Mechanical Engineering Congress & Exposition.

[5] Saeidi K., Akhtar F., Duriagina Z. (2018). Microstructure-Tailored Stainless Steels with High Mechanical Performance at Elevated Temperature. Stainless Steels and Alloys.

[6] Li J., Deng D., Hou X., Wang X., Ma G., Wu D., Zhang G. (2016). Microstructure and performance optimisation of stainless steel formed by laser additive manufacturing. Mater. Sci. Technol..

[7] Yakout M., Elbestawi M.A., Veldhuis S.C. (2018). On the characterization of stainless steel 316L parts produced by selective laser melting. Int. J. Adv. Manuf. Technol..

[8] Yadollahi A., Shamsaei N., Thompson S.M., Seely D.W. (2015). Effects of process time interval and heat treatment on the mechanical and microstructural properties of direct laser deposited 316L stainless steel. Mater. Sci. Eng. A.

[9] Barkia B., Aubry P., AGHI-Ashtiani P., Auger T., Gosmain L., Schuster F., Maskrot H. (2019). On the origin of the high tensile strength and ductility of additively manufactured 316L stainless steel: Multiscale investigation. J. Mater. Sci. Technol..

[10] Wang Y.M., Voisin T., McKeown J.T., Ye J., Calta N.P., Li Z., Zeng Z., Zhang Y., Chen W., Roehling T.T. (2018). Additively manufactured hierarchical stainless steels with high strength and ductility. Nat. Mater..

[11] Yin Y., Sun J., Guo J., Kan X., Yang D. (2019). Mechanism of high yield strength and yield ratio of 316 L stainless steel by additive manufacturing. Mater. Sci. Eng. A.

[12] Ma M., Wang Z., Wang D., Zeng X. (2013). Control of shape and performance for direct laser fabrication of precision large-scale metal parts with 316L Stainless Steel. Opt. Laser Technol..

[13] Kok Y., Tan X., Wang P., Nai M., Loh N., Liu E., Tor S. (2018). Anisotropy and heterogeneity of microstructure and mechanical properties in metal additive manufacturing: A critical review. Mater. Des..

[14] Everton S.K., Hirsch M., Stravroulakis P., Leach R.K., Clare A.T. (2016). Review of in-situ process monitoring and in-situ metrology for metal additive manufacturing. Mater. Des..

